# Breastfeeding and Snoring: A Birth Cohort Study

**DOI:** 10.1371/journal.pone.0084956

**Published:** 2014-01-08

**Authors:** Bronwyn K. Brew, Guy B. Marks, Catarina Almqvist, Peter A. Cistulli, Karen Webb, Nathaniel S. Marshall

**Affiliations:** 1 Woolcock Institute of Medical Research, Sydney, New South Wales, Australia; 2 The University of Sydney Medical School, Sydney, New South Wales, Australia; 3 Department of Medical Epidemiology and Biostatistics, Karolinska Institutet, Stockholm, Sweden; 4 Astrid Lindgren Children's Hospital, Lung and Allergy Unit, Karolinska University Hospital, Stockholm, Sweden; 5 Department of Respiratory and Sleep Medicine, Royal North Shore Hospital, Sydney, New South Wales, Australia; 6 Atkins Center for Weight and Health, University of California, Berkeley, California, United States of America; 7 The University of Sydney Nursing School, Sydney, New South Wales, Australia; Canadian Agency for Drugs and Technologies in Health, Canada

## Abstract

**Objective:**

To investigate the relationship between breastfeeding and snoring in childhood.

**Methods:**

In a cohort of children with a family history of asthma who were recruited antenatally we prospectively recorded data on infant feeding practices throughout the first year of life. Snoring status and witnessed sleep apnea were measured at age 8 years by parent-completed questionnaire. Associations were estimated by logistic regression with, and without, adjustment for sets of confounders designed to exclude biasing effects.

**Results:**

Habitual snoring was reported in 18.8% of the sample, and witnessed apnea in 2.7%. Any breastfeeding for longer than one month was associated with a reduced risk of habitual snoring at age 8 (adjusted OR 0.48, 95% CI 0.29 to 0.81) and duration of breastfeeding was inversely associated with the prevalence of habitual snoring (adjusted OR 0.79, 95% CI 0.62 to 1.00). Any breastfeeding for longer than 1 month was associated with a lower risk of witnessed sleep apnea (adjusted OR 0.17, 95% CI 0.04 to 0.71). The protective associations were not mediated by BMI, current asthma, atopy or rhinitis at age 8 years.

**Conclusions:**

Breastfeeding for longer than one month decreases the risk of habitual snoring and witnessed apneas in this cohort of children with a family history of asthma. The underlying mechanism remains unclear but the finding would be consistent with a beneficial effect of the breast in the mouth on oropharyngeal development with consequent protection against upper airway dysfunction causing sleep-disordered breathing.

## Introduction

Sleep disordered breathing (SDB) refers to a range of respiratory sleeping outcomes from primary snoring to obstructive sleep apnea (OSA). Although seemingly benign, in children SDB can be associated with hyperactivity, inattention, increased daytime sleepiness,[1]–[3] lower academic performance,[2], [4] high blood pressure,[5] and growth failure.[6] Risk factors for SDB in children include enlarged adenoids and/or tonsils, nasal allergy, frequent colds, prematurity, morphological features associated with a long narrow face and above average BMI.[7]–[9] Habitual snoring (more than 3 times per week) occurs in 7–22% of children,[1], [3], [10], [11] while OSA, which is characterized by abnormal pauses of breathing during sleep, occurs in approximately 2% of children.[11], [12] Both OSA and SDB occur in children of all ages from neonates to late adolescents [12], [13] although it is thought to be most common in 3–6 year olds when the size of the adenoid and tonsils is greatest compared to the throat diameter. [14]

Breastfeeding is an emerging protective factor against childhood snoring. The biological plausibility for this hypothesis comes via the mechanical effects of feeding from the breast, compared to a bottle, on the still plastic oral structures.[15] Two cohort studies have found that some breastfeeding reduces the risk of habitual snoring in children aged 1.5–6 years.[11], [16] However, other studies including our own have found that, breastfeeding for the recommended six months or more, compared to less than six months, was not associated with risk of habitual snoring in children aged 1 to 14 years.[10], [17]–[19] This suggests that breastfeeding in the first few months of life, rather than a long duration of feeding, may be important in preventing the development of habitual snoring in young children (critical timing of exposure). However, this hypothesis has not been tested.

The objective of this study was to investigate the relationship between infant feeding practices and SDB outcomes at age 8 years. Two specific aspects of infant feeding were studied: the duration of any breastfeeding and the duration of full breastfeeding (that is, consumption of no breastmilk substitutes, such as infant formula). The study sample is a well characterised cohort of mothers and children whose infant feeding practices were prospectively recorded in detail during the first twelve months of life.

## Methods

### Study population

The Childhood Asthma Prevention Study (CAPS) is a randomized controlled trial designed to test the effectiveness of house dust mite avoidance and fatty acid supplementation during the first five years of life as strategies for preventing asthma and allergy in children at high risk for asthma. Since asthma is a risk factor for SDB symptoms in children, this cohort is likely to have a relatively high prevalence of SDB symptoms, providing greater statistical efficiency to analyse associations. The study design, including the randomized interventions, and the outcomes of CAPS have been previously reported. [20]–[22] In brief, 616 pregnant mothers and their unborn babies were recruited between September 1997 and November 1999 from two hospitals in Sydney, Australia. The main eligibility criteria were that either parent or an older sibling had a history of asthma or recurrent wheeze, and that the child was born at >36 weeks gestation. Children born into households with pet cats were excluded. Six children who had been randomized were withdrawn for medical reasons immediately after birth. The presence of minor craniofacial abnormalities was not an exclusion criterion.

Data on the age of the child's mother, asthma status, smoking during pregnancy and both maternal and paternal educational levels, were recorded perinatally. Gestational age, birth weight and sex of the child were obtained from hospital records after birth. Smoking by any person inside the house was recorded biannually up to 8 years.

### Breastfeeding

Breastfeeding status was recorded by research nurses visiting the home at 1, 3, 6, 9 and 12 months. They recorded the age of the child when breastfeeding ceased, the age when breast milk substitutes were regularly given and whether the child had been given solids at 3, 6, 9 or 12 months. The breastfeeding variables were predetermined based on when the data was collected and the rate at which breast-milk substitutes and solid foods were introduced. *Fully breastfed for more than 3 months* was defined as no breast-milk substitute or solids before 3 months. *Any breastfeeding* was defined as any breast-milk given for at least one month regardless of whether other breast-milk substitutes or solids had been given. *Breastfeeding duration* was defined as the month at which breastfeeding ceased.

### Snoring

At age 8 years, parents filled in symptom questionnaires about asthma, eczema, allergy and sleep disorders including snoring. When the child was aged 8 years parents were asked “Does your child snore at present?” If they answered “Yes”, they were further asked “How often does your child snore?” with responses coded as “every night”, “more than three nights per week”, “more than once per week”, and “less than once per week”. For the purpose of these analyses snoring status was classified as both a dichotomous variable (*Any Snoring* Yes/No) and as an ordinal variable (5 categories; “None” through to “every night snoring”). *Habitual snoring* was defined as snoring for more than 3 nights per week. Loud snoring was defined as a response of “extremely loud”, “very loud” or “loud” to the question “How loud does your child snore?”. *Witnessed apnea* was defined by a positive response to “Does your child stop breathing in his/her sleep?” A range of categories from “less than once per week” to “stops breathing in his/her sleep every night” were further asked about, however, the low number of responses meant that we combined all positive responses and defined “witnessed apnea” as ever occurring. Difficulty breathing while sleeping was defined as a positive response to the question “Does your child struggle to breathe while sleeping?”

### Co-variates at age 8

*Current asthma* at age 8 years was defined as wheeze in the last 12 months and a doctor's diagnosis of asthma reported at any assessment up to and including 8 years. *Rhinitis (without cold)* was defined as rhinitis when not experiencing cold or flu symptoms. Height and weight were measured using a stadiometer and bathroom scales respectively. Body mass index (BMI) was calculated as weight (kg)/height (m)^2^.

### Statistical analysis

Descriptive statistics were calculated. Logistic regression was used to calculate the unadjusted and adjusted odds ratios. Where the outcome was dichotomous a simple logit model was used. The main effect, breastfeeding duration, was entered as an ordinal variable (<1 month, ≥1 to <7 months, ≥7 to <13 months and ≥13 months). The odds ratio for a one ordinal step increase in breastfeeding duration was estimated. It was not possible to include breastfeeding duration as a simple linear variable because data for exact duration was not available beyond 12 months. For some sub-groups it was not possible to compute an odds ratio because of zero values within a cell. In these cases, the significance of associations was estimated using Fishers exact test. For ordinal outcomes (eg frequency of snoring) a cumulative logit link function and multinomial error distribution were used to estimate odds ratios. The proportional odds assumption was tested to assess model validity.

Covariates were selected for inclusion in the models using a Directed Acyclic Graph (DAG) that were created using DAGitty Version 1.1 (Johannes Textor, Utrecht University, NL). [23] DAGS are a graphical based tool for making explicit the causal assumptions that underpin covariate selection and thereby do not rely on traditional epidemiological criteria for assessing potential confounders.[24] The following factors were considered in the context of the DAG: sex, birth weight, gestational age, mother's age at child birth, smoking in pregnancy, maternal education level, maternal asthma, environmental tobacco smoke (ETS) at 12 months of infant's age, ETS at 8 years, current asthma, rhinitis, atopy, and body mass index (BMI) at age 8 years. Relationships between each of the variables were assigned by BB and GM, based on knowledge of the literature regarding these associations. (Figure S1). Based on the assumptions described in the DAG,[23] we selected a set of adjustment variables (smoking in pregnancy, ETS at 8 yrs and maternal asthma) designed to minimise bias in the estimating the overall (“total”) effect of breastfeeding status on snoring. These variables are potential confounders in our model.

Potentially, some of the effects of breastfeeding on snoring may be mediated indirectly through intervening states, such as obesity, that are caused or prevented by breastfeeding and are, in turn, causal for snoring. In order to distinguish the effect of breastfeeding on snoring that is mediated indirectly in this way, from the direct effect of breastfeeding on snoring that is not mediated through identifiable intervening factors, we adjusted for these intervening causal factors in addition to the confounding factors. Based on the DAG, the adjustment set required to estimate this direct effect of breastfeeding on snoring was BMI at 8 years, ETS at 8 yrs, current asthma, birth weight, sex, smoking in pregnancy.

Analyses were performed using SAS Enterprise Guide 4.2 (SAS Inc, Chicago, IL, USA) and p≤0.05 was considered significant.

The study was approved by the human research ethics committees of the University of Sydney, the Children's Hospital at Westmead, and the Sydney South West Area Heath Services (Western Zone). Informed consent on behalf of the children was obtained from the parents in writing.

## Results

**Figure 1:**
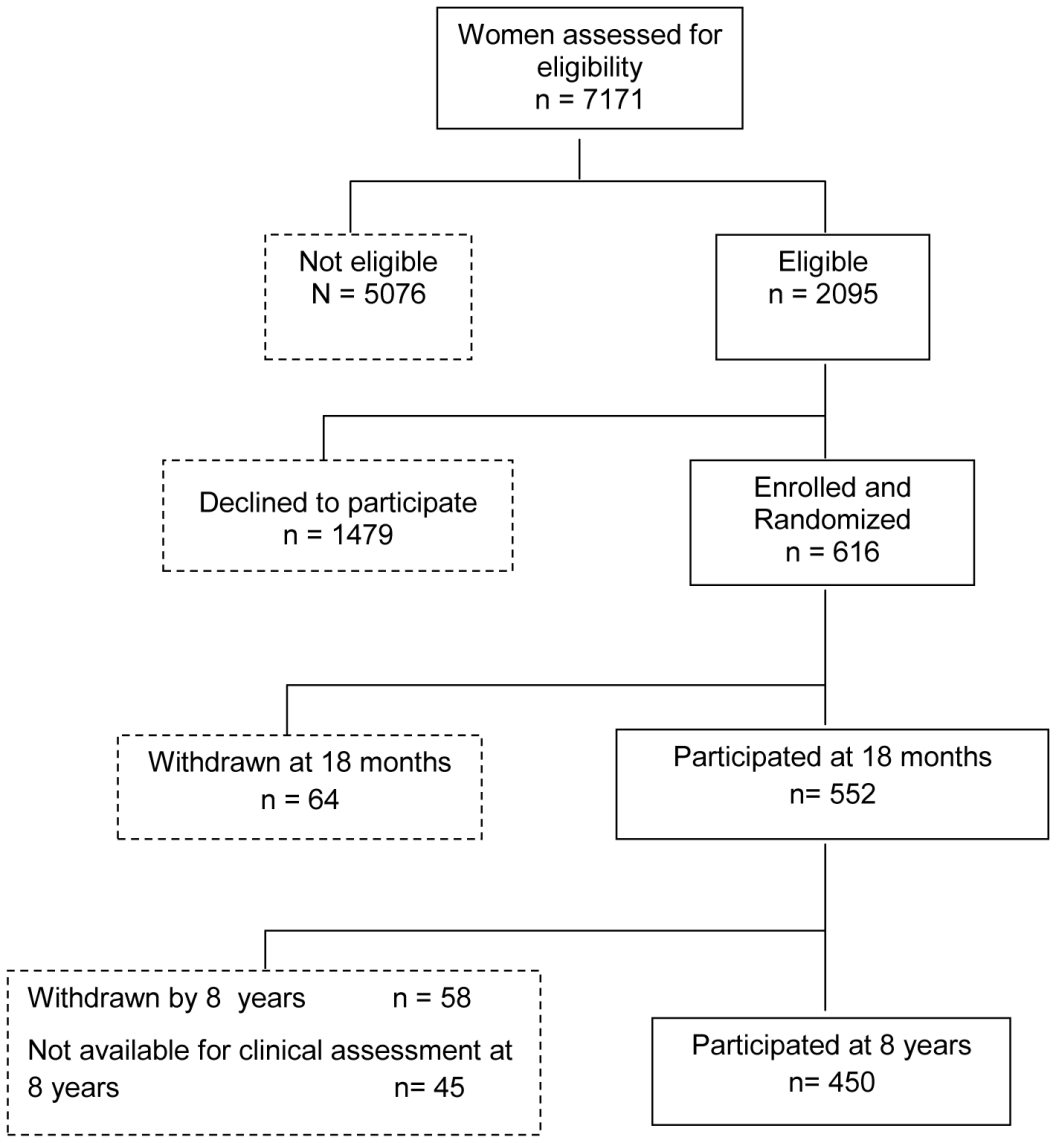
Flow chart displaying the participation in CAPS from birth to 8 years. Recruitment, entry and retention through 8 years of follow-up in the Childhood Asthma Prevention Study (CAPS) Trial.

Of the 616 participants recruited at birth, 64 withdrew from the study by 18 months, a further 58 withdrew by 8 years and a further 45 were not available for clinical assessment at 8 years. The final analyses at 8 years contained 450 (75%) of the original participants (Figure 1).

Table 1 displays the baseline characteristics of the original cohort and those remaining in the study at age 8 years. The characteristics of the study population available for this analysis do not differ from those of the original cohort except that the parents of those remaining in the study at 8 years had a higher education level than those in the original cohort.

**Table 1:** Characteristics of CAPS subjects assessed at 8 years and of the original cohort recruited antenatally.

		Mean ± SD or n (%)	Mean ± SD or n (%)
		Subjects remaining at 8 yrs	Birth Cohort
		N = 450	N = 616
**Breastfeeding**			
At least 3 months full breastfeeding		144 (33.9)	170 (30.6)
Any breastfeeding for at least one month		313 (69.6)	389 (67.1)
Breastfeeding duration in months			
	<1	137 (30.4)	191 (32.9)
	≥1 to <7	149 (33.1)	191 (32.9)
	≥7 to <13	78 (17.3)	94 (16.2)
	≥13	86 (19.1)	104 (17.9)
**Sex** (male)		228 (50.7)	312 (50.7)
**Gestational age** (wks)		39.58±1.26	39.56±1.26
**Birth weight** (kg)		3.50±0.49	3.49±0.49
**Mother's age at child's birth** (yrs)		29.52±5.08	28.97±5.28
**Number of Older Siblings**			
	0	151 (33.6)	199 (32.6)
	1	156 (34.7)	214 (35)
	2	89 (19.8)	129 (21.1)
	≥3	53 (11.8)	69 (11.3)
**Maternal Asthma**		242 (53.8)	347 (56.3)
**Mother smoked in pregnancy**		106 (23.6)	150 (24.4)
**ETS in home at age 1** (more than one cigarette smoked/day)		125 (27.8)	165 (29.5)
**Mother's education**			
	University/TAFE	223 (49.6)	276 (44.8)
	Year 12	78 (17.3)	110 (17.9)
	Year 10	133 (29.6)	207 (33.6)
	≤Year 9	16 (3.6)	23 (3.7)
**Covariates at age 8**			
**ETS in home at 7.5 yrs**		80 (18.4)	
**Current asthma at 8 yrs**		98 (21.8)	
**Rhinitis (without cold) 8 yrs**		121 (27.0)	
**BMI at 8 yrs kg/m^2^**		17.49±2.98	
**Atopy at 8 yrs (positive to any SPT)**		181 (45.0)^a^	

^a^ Not all children agreed to undergo a skin prick test, n = 402.

Parents reported that 179 (39.8%) children were snoring at 8 years of age. Fifty four children (12%) snored every night, 84 (18.8%) children were habitual snorers and 21 (4.7%) children were reported as being ‘loud’ snorers. Twelve children (2.7%) had witnessed apneas and 31 (6.9%) were observed struggling to breathe while sleeping.

Parent reported snoring was less common in children who were fully breastfed for more than three months than those who were fully breastfed for less than three months (Table 2). The protective effect was only statistically significant for habitual snoring and, although adjustment for potential confounders made little difference to the estimated odds ratio, the 95% confidence intervals included 1.0 after adjustment. No child who had been fully breastfed for longer than three months had witnessed apnea (p = 0.02 compared with those who had been fully breastfed for less than three months).

**Table 2:** Full Breastfeeding for more than 3 months and Snoring Outcomes.

	Prevalence (%) of Snoring outcome/Breastfeeding category			TOTAL EFFECT	DIRECT EFFECT
	Full Breastfeeding ≥3 months	Full Breastfeeding <3 months	Unadjusted OR (95% CI)	Adjusted OR^c^ (95% CI)	Adjusted OR^d^ (95% CI)
	(n = 144)	(n = 281)	N = 425^a^	N = 410^b^	N = 410^b^
**Any Snoring (n = 170)**	34.0	43.1	0.68 (0.45, 1.04)	0.77 (0.5, 1.20)	0.84 (0.53, 1.33)
**Habitual Snoring (n = 80)**	13.2	21.7	0.55 (0.31, 0.96)	0.61 (0.34, 1.10)	0.61 (0.33, 1.14)
**Loud Snoring (n = 19)**	2.8	5.3	0.51 (0.17, 1.56)	0.49 (0.16, 1.55)	0.48 (0.13, 1.78)
**Difficulty breathing while Sleeping (n = 27)**	4.9	7.1	0.67 (0.28, 1.62)	0.76 (0.30, 1.91)	0.87 (0.31, 2.42)
**Witnessed Apnea (n = 11)**	0	3.9	NA	NA	NA

Odds Ratios (OR) and 95% Confidence Intervals (CI).

^a^ .Information on Full breastfeeding status was missing for 25 participants ^b^.Information on covariates was missing for 15 subjects who were excluded from the adjusted models ^c^.Adjusted for ETS at 7.5 yrs, maternal asthma, smoking in pregnancy ^d^.Adjusted for BMI (8 yrs), ETS at 7.5 yrs, current asthma, birth weight, sex, smoking in pregnancy, rhinitis at 8 years.

**Figure 2:**
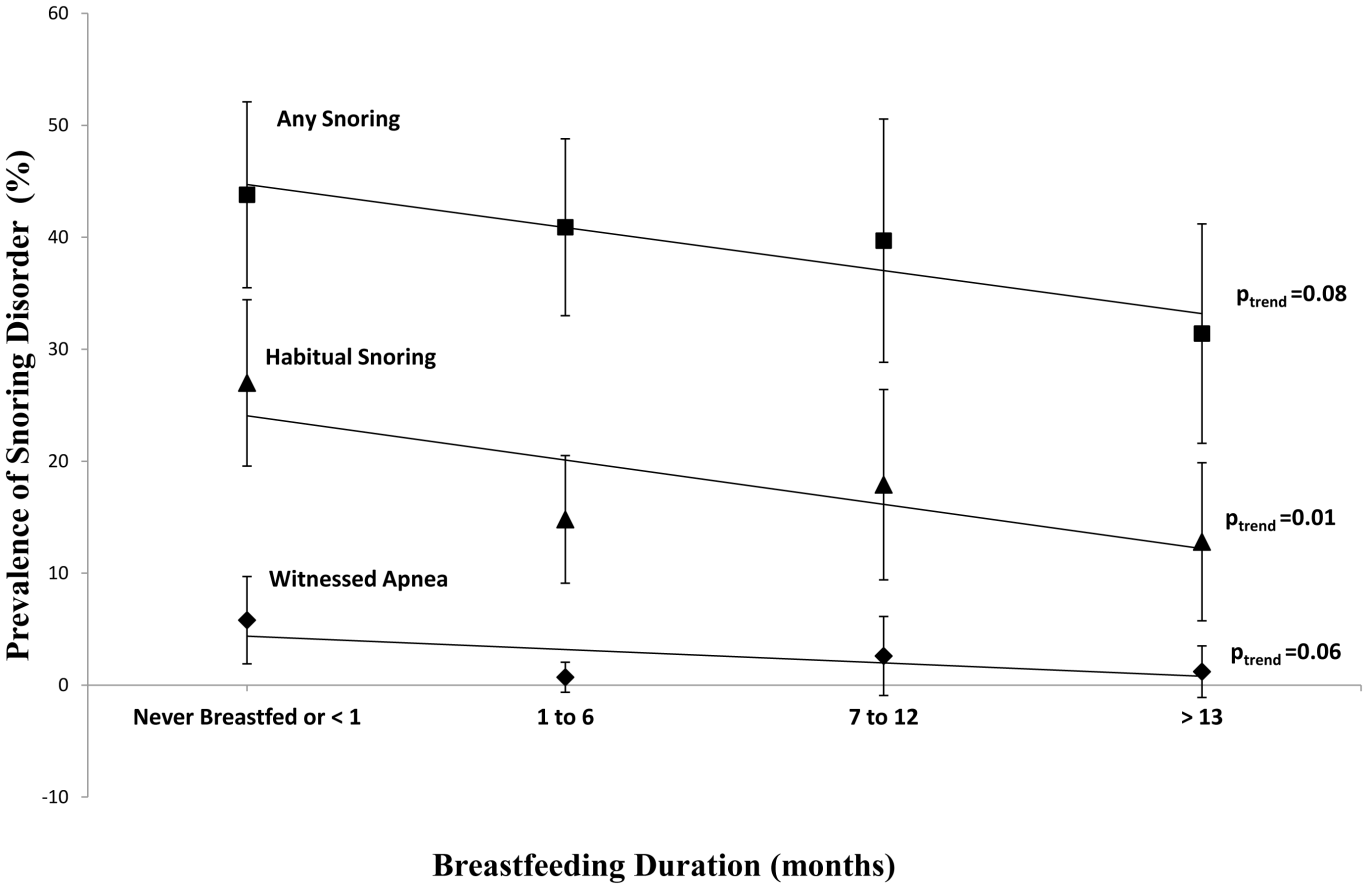
Associations between Breastfeeding Duration and Snoring outcomes. CAPS cohort N = 450. Comparing the prevalence of different parent reported sleep disordered breathing at age 8 against the amount of breastfeeding recorded during the first year of life to see whether breastfeeding has a dose-response relationship with protection against later snoring or sleep apnea. P-values indicate significance of a linear trend and the error bars are 95% confidence limits for the proportions.

Any breastfeeding for longer than a month, compared with less than one month, was associated with a reduced risk for both habitual snoring (OR_adj_ 0.48, 95% CI 0.29, 0.81) and witnessed apnea at age 8 (OR_adj_ 0.17, 95%CI 0.04, 0.71, see table 3). There was a dose-response relationship between duration of breastfeeding and the presence of habitual snoring at age 8 years (Table 4, Figure 2). The adjusted odds ratio for habitual snoring for each step increase in breastfeeding duration was 0.79, 95% CI 0.62, 1.00. Although any reported snoring and witnessed apneas tended to be less common with increasing breastfeeding duration, this trend was not significant (Figure 2, Table 4).

**Table 3:** Breastfeeding for more than a month and Snoring Outcomes.

	Prevalence (%) of Snoring outcome/Breastfeeding category			TOTAL EFFECT	DIRECT EFFECT
	Any Breastfeeding ≥1 month	Any Breastfeeding Never or <1 month	Unadjusted OR (95% CI)	Adjusted OR^b^ (95% CI)	Adjusted OR^c^ (95% CI)
	(n = 313)	(n = 137)	N = 450	N = 416^a^	N = 416^a^
**Any Snoring (n = 179)**	38.0	43.8	0.79 (0.52, 1.18)	0.84 (0.55, 1.29)	0.86 (0.55, 1.35)
**Habitual Snoring (n = 84)**	15.0	27.0	**0.48 (0.29, 0.78)**	**0.48 (0.29, 0.81)**	**0.48 (0.28, 0.82)**
**Loud Snoring (n = 21)**	3.8	6.6	0.57 (0.23, 1.38)	0.56 (0.22, 1.39)	0.67 (0.25, 1.76)
**Difficulty breathing while Sleeping (n = 31)**	7.0	6.6	1.07 (0.48, 2.4)	1.09 (0.48, 2.50)	1.13 (0.47, 2.72)
**Witnessed Apnea (n = 12)**	1.3	5.8	**0.21(0.06, 0.71)**	**0.17 (0.04, 0.71)**	**0.22 (0.05, 0.91)**

Odds Ratios (OR) and 95% Confidence Intervals (CI).

^a^ .Information on covariates was missing for 34 subjects who were excluded from the adjusted models ^b^.Adjusted for ETS at 7.5 yrs, maternal asthma, smoking in pregnancy ^c.^Adjusted for BMI (8 yrs), ETS at 7.5 yrs, current asthma, birth weight, sex, smoking in pregnancy, rhinitis at 8 years.

**Table 4:** Breastfeeding Duration in categories and Snoring Outcomes.

	Prevalence (%) of snoring outcome/breastfeeding duration						
	Never breastfed or <1 month	1–6 months breastfeeding	7–12 months breastfeeding	>13 months breastfeeding	Trend	Unadjusted OR (95% CI)	Adjusted OR^b^ (95% CI)
	(n = 137)	(n = 149)	(n = 78)	(n = 86)		N = 450	N = 416^a^
**Any Snoring (n = 179)**	43.8	40.9	39.7	31.4	p = 0.08	0.85 (0.72, 1.02)	0.90 (0.75, 1.09)
**Habitual Snoring (n = 84)**	27.0	14.8	17.9	12.8	p = 0.01	0.75 (0.59, 0.95)	0.79 (0.62, 1.00)
**Loud Snoring (n = 21)**	6.6	3.4	5.1	3.5	p = 0.38	0.83 (0.54, 1.26)	0.83 (0.54, 1.29)
**Difficulty Breathing while sleeping (n = 31)**	6.6	8.7	5.1	5.8	p = 0.60	0.92 (0.65, 1.30)	0.95 (0.66, 1.35)
**Witnessed Apnea (n = 12)**	5.8	0.7	2.6	1.2	p = 0.07	0.54 (0.28, 1.05)	0.56 (0.27, 1.17)

Odds Ratios (OR) and 95% Confidence Intervals (95%CI).

^a^ .Information on covariates was missing for 34 subjects who were excluded from the adjusted models ^b^.Adjusted for ETS at 7.5 yrs, maternal asthma, smoking in pregnancy.

**Figure 3:**
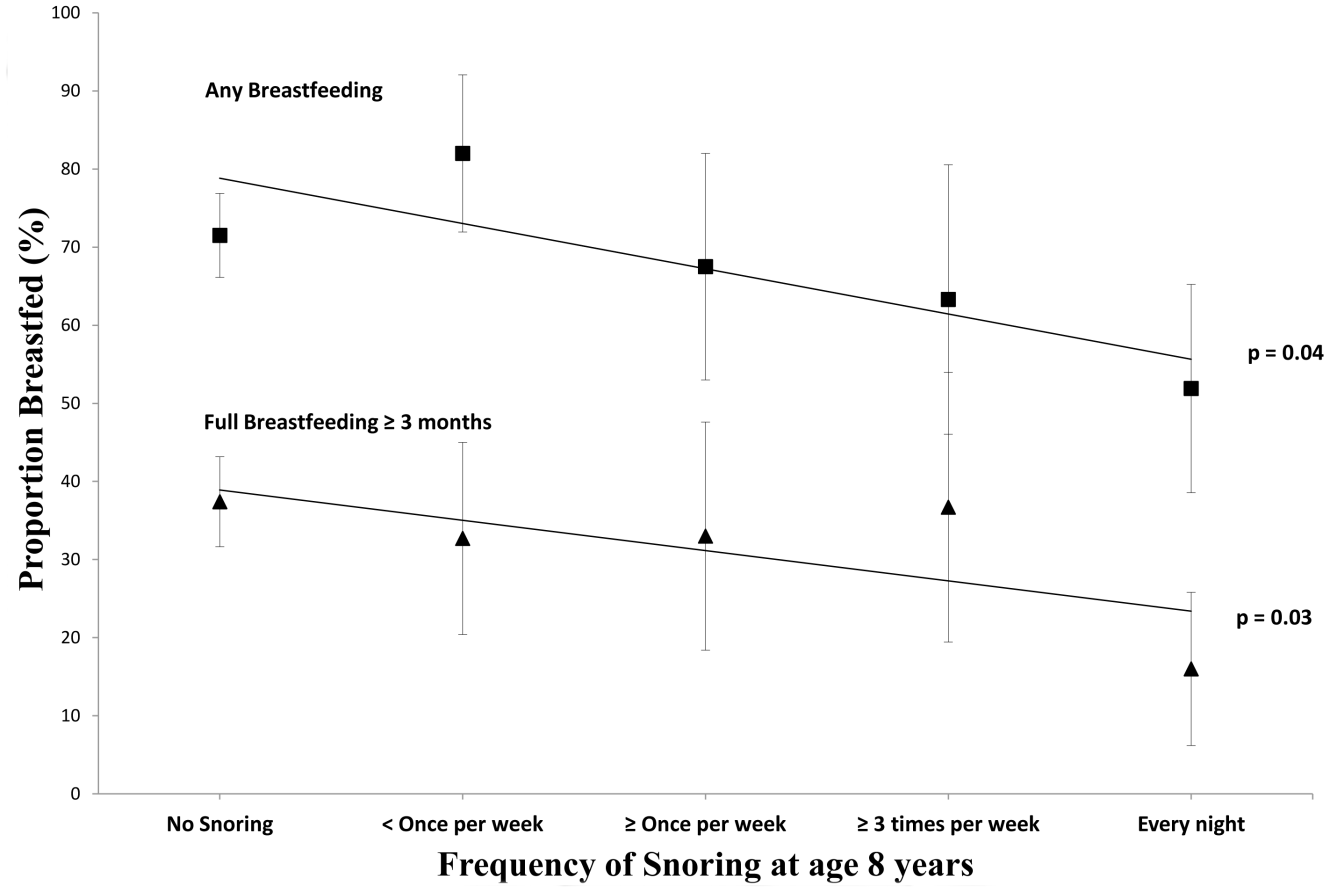
Associations between Frequency of Snoring at 8 years and the Proportion of those Breastfed. CAPS cohort N = 450. Comparing the prevalence of different definitions of breastfeeding in the first year of life with parentally-reported snoring frequency at age 8 to see whether there may be a dose-response relationship between breastfeeding and protection against snoring in middle childhood. P-values indicate the significance test for the ordinal odds ratio and the error bars are 95% confidence limits for the proportions.

Figure 3 shows that the frequency of reported snoring at age 8 years is inversely related to the prevalence of being breastfed for longer than a month or fully breastfed for longer than 3 months. The unadjusted odds ratios for a one step increase in snoring frequency associated with having been breastfed for longer than a month or fully breastfed for longer than three months were 0.66 (95% CI 0.45 to 0.98) and 0.63 (95% CI 0.42 to 0.95) respectively. When confounders were included in this multinomial logistic model the POA was not sustained and therefore we were unable to determine the adjusted odds ratio.

The total and direct effect odds ratios for the association between breastfeeding and parent reported snoring were very similar. This suggests that the associations were not mediated via any potential intervening factors on the causal pathway between breastfeeding and snoring, such as obesity or asthma.

## Discussion

This study has shown that breastfeeding for more than one month is associated with a reduced risk for parent reported habitual snoring and witnessed apneas at 8 years of age. This association persists after adjustment for potential confounding factors. Longer duration of breastfeeding in the first year of life is associated with greater reduction in the risk of parent reported habitual snoring and witnessed apneas reported at age 8 years. However, full breastfeeding for more than 3 months, compared to less than 3 months, had no association with parent reported habitual snoring.

The main strength of this study is that infant feeding practices were recorded contemporaneously during the first year of life, thereby reducing the risk of recall bias. Furthermore, we have recorded data that allows us to quantify the duration of both any breastfeeding and full breastfeeding during the first year of life. This is an advantage over other studies which have only analysed breastfeeding in a binary manner, either any or none, or more or less than 6 months. In addition, other measurements used in our adjusted analyses were recorded concurrently, either prenatally e.g. smoking during pregnancy or at the time of the 8 year assessments e.g. environmental tobacco smoke at 7.5 years, BMI at 8 years. Another strength of this study is the use of the DAG assessment of covariates. This method gives us confidence in the causal inferences derived from the analysis and allows us to distinguish direct effects of breastfeeding on snoring from those that are mediated via the effect of breastfeeding on intervening factors.[23] The fact that the total and direct effects were similar suggests that the protective effect of breastfeeding on snoring is not mediated via an effect on obesity or asthma status. Although we acknowledge that an observational study cannot prove causality of a risk factor on an outcome, it is unethical to use a randomized control trial for breastfeeding studies, therefore, cohort studies adjusting for confounders are a feasible way to test these associations.

The main limitation of this study is the lack of objective measures of snoring and SDB. The outcomes in this study were measured by parental report of observed symptoms and signs. Although the optimal method for measuring snoring and sleep apnea is laboratory-based polysomnography, this was not feasible in our cohort study focussed on allergic diseases. However, Montgomery-Downs et al have shown that parental report of frequent snoring is a valid indicator when assessed against polysomnography.[25] Furthermore, our sleep apnea prevalence estimates were similar to other child population studies, although snoring was more common.[11], [12]

Another key limitation is that the breastfeeding exposure categories have been measured in a manner that is relevant to the immunological hypothesis that breast-milk itself protects against asthma.[26] For this reason, we recorded information on breast-milk feeding, rather than the mode of feeding. Therefore, those children who were classified as *fully breastfed for more than 3 months* may have received some or all of their milk as expressed breast-milk from a bottle during that time.[27], [28] The effect of this potential misclassification would be to attenuate the observed association toward the null. Hence, the associations we observed between absence of full breastfeeding and SDB symptoms may actually be an underestimate of the true effect. Unfortunately we did not have reliable information about whether any children had a tonsillectomy/adenoidectomy, an operation which may have cured some children of their SDB symptoms. If some of those children with snoring did have this operation this would also tend to attenuate the observed associated.

We also observed that the prevalence of habitual snoring in this cohort was higher than in other studies conducted in children of a similar age. [1], [10], [11] This could be because the children have a family history of asthma. The fact that all of children in this cohort had a family history of asthma limits the generalisability of our findings. However, our results are in agreement with other studies in this field [11], [16], [26] and therefore can be used to support the body of evidence on this subject.

It is noteworthy that none of the 12 children who had witnessed apneas reported had been fully breastfed for longer than 3 months. Although this seems to imply a protective effect of fully breastfeeding for three months or more against witnessed apneas, in the absence of data on orofacial abnormalities in infancy, we cannot exclude the possibility that these children had pre-existing minor anatomical abnormalities that made breastfeeding difficult and also predisposed them to sleep apnea. Babies with serious health problems were excluded from the cohort, therefore the probability of having orofacial abnormalities is reduced but not removed. This cohort did not include pre-term infants and therefore we have not assessed the impact of breastfeeding in pre-term infants.[9]

The results of our study are similar to those of the Avon Longitudinal Study of Parents and Children (ALSPAC) which found that any breastfeeding compared with none was associated with a reduced risk for always snoring and habitual snoring from 1.5 to 6.75 years of age.[11] Our results extend this knowledge by showing that the longer a child is breastfed the less likely they will have habitual or frequent snoring in mid childhood. Other studies have compared more and less than 6 months of breastfeeding and found no difference in the risk of snoring.[10], [19] Hence, six months is probably not the critical duration of breastfeeding for achieving protection against snoring. Our data, together with data from previous published studies, [11] suggest that breastfeeding for greater than one month but less than 6 months is sufficient to reduce the risk of SDB symptoms.

It has been suggested that breastfeeding may protect against snoring and sleep apnea, an effect that is mediated by the physical effect of the breast in the mouth on oropharyngeal development.[15] Indeed, there is some evidence that breastfeeding has a beneficial effect on mandible development [29], [30] and causes less malocclusions when compared to bottle feeding. [31]–[33] Further, several cross-sectional studies have used cephalometry and orthodontic casts to establish associations between facial shape and snoring and OSA in children. A long, narrow face (dolichocephaly), [8] narrow palate, [8], [17], [34] overcrowding of teeth,[8] maxillary constriction with cross-bite, [8], [17] overjet, [34] and deep palatal height [17] are each associated with upper airway obstruction and sleep disordered breathing. These same craniofacial abnormalities are observed in adults with snoring and OSA, and it is thought that these evolve during childhood development. [35] Therefore, it is biologically plausible that if breastfeeding affects orofacial development during the first few months of life this could have an effect on whether the child has an anatomical predisposition to SDB. Montgomery-Downs has also proposed an immunological hypothesis to explain the association between breastfeeding and snoring; breastfeeding provides immunoglobulins that help prevent infection from respiratory viruses therefore decreasing potential inflammation that may lead to sleep disordered breathing.[26] Another hypothesis is that the *suckling* that occurs with breastfeeding (in contrast to the *sucking* action associated with bottle-feeding) is associated with a peristaltic motion of the tongue underneath the breast, and this allows the proper development and co-ordination of oropharyngeal musculature required for swallowing.[36]

This study found that breastfeeding for longer than one month and full breastfeeding for longer than 3 months significantly decreased the risk of witnessed sleep apnea at age 8 years. These results differ from the ALSPAC study which did not find any association between breastfeeding and sleep apnea at any age.[11] However, our findings support a clinically recruited study of snoring children where sleep was objectively measured via polysomnography and where breastfeeding was retrospectively collected. Breastfeeding for at least two months was associated with reduced severity of SDB on all measures employed.[26]

In conclusion, breastfeeding for longer than one month is associated with a reduced risk of parent reported habitual snoring and witnessed apneas. No child in our study who was fed breastmilk only for longer than three months had witnessed apneas at age 8. Our data suggest that breastfeeding for between one and six months was sufficient to protect children from the apparent adverse effects of bottle-feeding on the risk of SDB symptoms.

## Supporting Information

Figure S1 **Directed Acyclic Graph for assessing the Causal association between Breastfeeding and Snoring.** green circle =  *exposure* blue circle =  *outcome* blue oval =  *ancestor of outcome*. grey oval =  *unobserved (latent)* pink oval =  *ancestor of exposure and outcome*. green line =  causal path pink line =  biasing path.
(TIF)
