# Supplementary figures and images for: Breastfeeding and Snoring: A Birth Cohort Study
Source: PLoS One. 2014 Jan 8;9(1):e84956. doi: 10.1371/journal.pone.0084956 (PMC3885662; doi:10.1371/journal.pone.0084956)

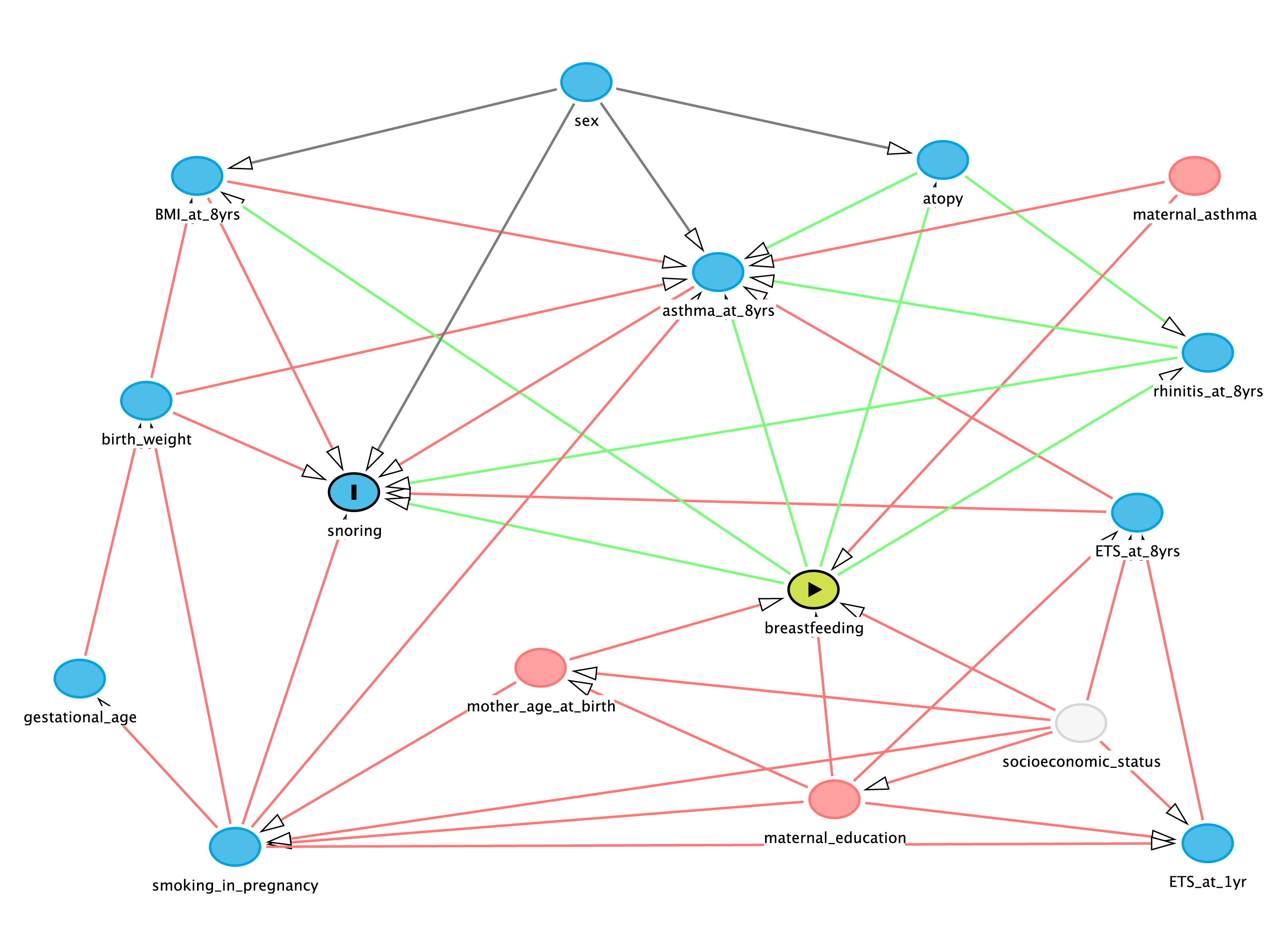

Supplement: Figure S1 — Directed Acyclic Graph for assessing the Causal association between Breastfeeding and Snoring. green circle = exposure blue circle = outcome blue oval = ancestor of outcome. grey oval = unobserved (latent) pink oval = ancestor of exposure and outcome. green line = causal path pink line = biasing path. (TIF) [file pone.0084956.s001.tif]
